# Ex Vivo Differentiation of Cartilage Degeneration Severity and Subchondral Bone Using Vibroacoustic Signals from Instrument–Tissue Interaction

**DOI:** 10.3390/s26154874

**Published:** 2026-08-02

**Authors:** Thomas Sühn, Maximilian Costa, Nazila Esmaeili, Moritz Spiller, Axel Boese, Jessica Bertrand, Michael Friebe, Alfredo Illanes, Christoph H. Lohmann

**Affiliations:** 1 Department of Orthopaedic Surgery, Otto-von-Guericke University Magdeburg, 39120 Magdeburg, Germany; 2 SURAG Medical GmbH, 04229 Leipzig, Germany; 3 Center for Digital Surgery, Department of General, Visceral and Pediatric Surgery, University Medical Center Göttingen, 37075 Göttingen, Germany; 4 INKA Innovation Laboratory for Image Guided Therapy, Otto-von-Guericke University Magdeburg, 39120 Magdeburg, Germany; 5 Faculty of Computer Science, AGH University of Krakow, 30-059 Kraków, Poland; 6 Faculty of Computer Science, Otto-von-Guericke University Magdeburg, 39120 Magdeburg, Germany; 7 Research Campus STIMULATE, Otto-von-Guericke University Magdeburg, 39120 Magdeburg, Germany

**Keywords:** knee osteoarthritis, articular cartilage, intraoperative tissue characterization, vibroacoustic sensing, machine learning, continuous wavelet transform, surgical data science, computer-assisted surgery, decision support, knee arthroplasty

## Abstract

Osteoarthritis (OA) of the knee is characterized by cartilage matrix degeneration, which eventually leads to joint dysfunction and surgical replacement. Preoperative radiography, the standard for treatment decision making, provides limited information on the extent of degeneration, emphasizing the need for quantitative intraoperative techniques. This study evaluates the potential of vibroacoustic signals generated from instrument–tissue interactions to assess cartilage degeneration severity. A total of 136 ex vivo cartilage specimens of varying degeneration severity, histologically graded using the OARSI score, were collected from 41 patients undergoing arthroplasty. The specimens were classified into three groups: healthy cartilage (OARSI ≤2.0), degenerated cartilage (OARSI ≥ 2.5), and subchondral bone. Vibroacoustic signals were captured during specimen palpation using a vibration measurement system affixed to a surgical probe. 26 characteristic signal features were extracted using Continuous-Wavelet Transformation, and their discriminative power was assessed using Support Vector Machine and k-Nearest-Neighbor classifiers under patient- and specimen-level grouped cross-validation with nested hyperparameter tuning. Bone was distinguished from cartilage with 84–91% recall. Healthy cartilage (OARSI ≤2.0) was identifiable with 74–80% sensitivity, whereas the specificity for degenerated cartilage (OARSI ≥ 2.5) remained limited (40–57%), constituting the principal limitation of the approach. Interpreting the binary cartilage classification as a diagnostic test, a receiver-operating characteristic (ROC) analysis yielded an area under the curve (AUC) of up to 0.66 (95% CI [0.56, 0.76]). The study demonstrates that vibroacoustic signals from instrument–tissue interactions may provide complementary information for intraoperative cartilage evaluation and may contribute to decision making in arthroplasty, while the reliable grading of cartilage degeneration remains an open challenge.

## 1. Introduction

Osteoarthritis (OA) of the knee is one of the most frequent joint diseases of the elderly population, with approximately 15% of the world’s population suffering from OA [1]. The main symptom of this disease is pain, leading to joint dysfunction and activity limitation in adults [2]. The standard diagnosis of OA includes patient history and physical examination, followed by various imaging modalities of the joint, such as radiography. Typical radiographic signs of OA are osteophytes, joint space narrowing, thickening of the subchondral bone, and occasional bone cysts. While plain radiographs do not reveal minor cartilage changes, especially in cases of early OA [3], it is still the primary diagnostic gold standard for OA using the Kellgren–Lawrence Score [4,5]. A study comparing preoperatively and intraoperatively assessed Kellgren–Lawrence Scores has shown that the score is inaccurate in describing the disease severity [6]. Further, the score varies widely within an inter-observer group due to the highly subjective nature of this diagnosis [7]. However, it is used as the primary decision-making tool to determine the need for endoprosthesis implantation in clinical daily routines [8]. In the clinical setting, knowledge about cartilage quality can be a valuable basis for treatment decisions, such as an indication for unilateral knee arthroplasty (UKA) instead of total knee arthroplasty (TKA) [9]. The potential advantages of UKA over TKA are reduced blood loss, quicker patient rehabilitation, and better postoperative functionality of the joint [10,11].

In contrast to radiography, the histological OARSI (Osteoarthritis Research Society International) score is a reliable system for grading osteoarthritic cartilage [12]. On safranin-orange/fast-green-stained sections, it grades surface fissures, cell proliferation, cell death, and proteoglycan loss, ranging from an intact surface (score 0) through early fissuring and softening (score 1) [13] to complete cartilage loss with subchondral bone exposure (score 6). However, the drawback of any histological analysis is its dependency on a tissue specimen and typically a complex and time-consuming preparation and analysis of the tissue. Further, collecting cartilage specimens is associated with an invasive intervention and inevitable damage to the cartilage surface at the sample location. In contrast to radiography, this makes it unsuitable for any intraoperative and non-destructive in vivo assessment of the OA cartilage. While both methods mentioned are based on visual inspection of images, the structural changes related to OA are also reflected in the mechanical behavior of the cartilage [14,15]. This fact can be utilized for sensor-based technologies with direct cartilage contact and the potential for an intraoperative assessment. The need and potential advantage of such an assessment have led to the exploration of new modalities such as Near-Infrared Spectroscopy (NIRS) [16,17,18,19], Optical Coherence Tomography (OCT) [20,21], indentation-type atomic force microscopy (IT-AFM) [22,23,24] and Fiber Bragg Grating (FBG)-based micro-indentation [25,26,27]. While these technologies show high potential in the lab environment and ex vivo tissue specimens, they have not yet been adopted in clinical routines or even certified. Primary barriers are the need for additional expensive devices (NIRS, OCT, IT-AFM), the challenge of integration into arthroscopic instruments (FBG), and the integration into the surgical workflow and setup.

In the current clinical routine, orthopaedic surgeons must mainly rely on preoperative radiographs and intraoperative localization, visual assessment, and physical inspection of the degenerated cartilage surface. This inspection can be done via manual palpation in the case of open UKA or TKA or via a palpation probe in the case of arthroscopic surgery. It is highly dependent on the examiner’s experience, causing high variations in the result [28]. To mitigate some of the challenges related to intraoperative assessment, we propose a computerized vibroacoustic palpation of the cartilage surface. Here, the structural vibrations generated during the interaction between the palpation probe and cartilage are used as the source of information. The feasibility of this concept has been shown for different minimally invasive instruments and procedures such as needle interventions [29,30], laparoscopic surgery [31], or a potential application in robot-assisted surgery [32,33]. In the present work, we investigated the capability of computerized palpation to differentiate between healthy cartilage, degenerated OA cartilage, and subchondral bone based on vibroacoustic signals. Biological tissues such as cartilage, tendon, and bone have been shown to exhibit individual vibroacoustic palpation signatures, from which characteristic features can be extracted for machine-learning-based discrimination [34,35].

In contrast to these earlier feasibility studies on coarse tissue-type discrimination, the present work targets the substantially harder problem of grading the severity of degeneration within a single tissue type and frames the binary cartilage decision as a diagnostic test, which is the methodological contribution of this study. We hypothesize that (1) due to OA cartilage’s structural and bio-mechanical changes, palpation-based features can be extracted for different degrees of degeneration; and (2) that these can be used for differentiation purposes. This study aims to prove the approach’s feasibility as a basis for a complementary, intraoperative cartilage quality assessment in the arthroplasty use case.

## 2. Materials and Methods

### 2.1. Patient Cohort and Ex Vivo Specimen Preparation

Ex vivo tissue specimens of OA cartilage with differing grades of degeneration were collected from patients undergoing UKA or TKA at the Department of Orthopaedic Surgery of the University Hospital Magdeburg. Informed written consent was obtained from the patients before surgeries. Ethical approval for the study was obtained from the Institutional Review Board of the Medical School of Otto-von-Guericke University (No 55/20) and all methods were performed in accordance with the relevant guidelines and regulations. A total of 41 patients were included, and patient data, radiographic images, and other diagnostic results were pseudonymized and incorporated into a database (Table 1). Exclusion criteria were inflammatory joint conditions such as diagnosed rheumatic diseases or infections. Portions of the femoral condyles removed during the surgeries were collected for this study and immediately preserved in phosphate-buffered saline (PBS, composition 137 mM NaCl, 2.7 mM KCl, 1.4 mM Na_2_HPO_4_, 1.4 mM KH_2_PO_4_, pH value = 7.4). The tissues remained in this buffer solution for an average of 49 min before further processing. The condyles can comprise regions of highly preserved cartilage, different degrees of OA, and plain subchondral bone due to total cartilage loss. For specimen preparation, the condyles were removed from the PBS, and an orthopedic surgeon visually identified up to 4 regions of interest. Specimens with a size of 10×5×5 mm comprising the cartilage and part of the subchondral bone were cut using a punch and put into PBS before data acquisition. A total of 138 ex vivo cartilage specimens from 43 knees were obtained, of which 136 specimens from 41 knees entered the analysis after excluding cases with incomplete histological grading or signal data.

### 2.2. Computerized Palpation

**Figure 1:**
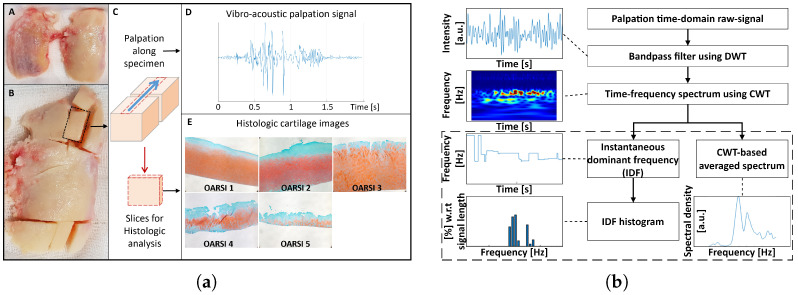
Steps for cartilage specimen preparation and data acquisition (**a**) and signal processing for feature extraction (**b**). (**a**) A: Femoral condyle. B: Lateral condyle with specimen obtained using a punch. C: Illustration of the computerized palpation path and the histological slice. D: Resulting vibroacoustic palpation signal. E: Resulting histologic images of OARSI scores 1–5. (**b**) Signal processing steps performed to derive three indicators: instantaneous dominant frequency (IDF), IDF histogram, and CWT-based stationary spectrum, from which 26 characteristic features were extracted.

The approach uses vibrations resulting from the interaction between the palpation probe and tissue surface as a source of information. The interactions generate vibroacoustic waves propagating naturally along the instrument to the opposite proximal end. This vibroacoustic signal can be acquired from the instrument’s surface, and information related to the interacting tissue surface can be extracted. For the data acquisition, a wireless vibration measurement system (SURAG Medical GmbH, Leipzig, Germany) was mounted to a typical palpation probe (28124 BZ, KARL STORZ SE & Co. KG, Tuttlingen Germany). The stainless steel rod, with a 23 mm length, 2.7 mm diameter, and rounded cone-shaped tip, is used in arthroscopy to examine anatomical structures within the knee [36]. The measurement system incorporates a mechano-acoustic configuration described in [33] to amplify the vibroacoustic signal before acquisition with an acoustic transducer. After analog signal conditioning, a quantization with a sampling frequency of $f_{s}=16$ kHz and 18 Bits resolution is performed. The signals are stored as audio files. The computerized palpation was performed by six volunteers who had experience in laboratory experiments and tissue specimen handling. Each volunteer received supervised training on the measurement system and the protocol for the data acquisition. After moisturizing the specimen in PBS for 1 s, it was mounted in a holding frame to ensure a stable position during palpation. Then, a volunteer performed a measurement defined as 20 consecutive palpations in a defined direction and over the 10 mm length of the specimen. A contact angle of ≈45° between the instrument tip and the specimen surface was used. The resulting ≈30 s long audio file contained 20 palpations, each defined as an individual experiment. Per specimen, a minimum of two to four volunteers performed the measurement consecutively and without interruption. Subsequently, the specimen was placed in PBS for the following histological analysis. Figure 1 depicts the femoral condyles collected during surgery, the specimen obtained, and examples of a vibroacoustic palpation signal and histologic images as the basis for the analysis.

### 2.3. Histological Analysis

For each specimen, a full-thickness cartilage sample of the palpated region was dissected from the bone for further paraffin embedding. The cartilage samples were fixed in 4% paraformaldehyde in PBS at 4 °C for 24 h. Subsequently, the samples were dehydrated through a graded series of ethanol solutions and embedded in paraffin. 5 μm slices were cut, deparaffinized, and stained for histochemistry. Safranin-orange staining was applied, and histological images were created to evaluate osteoarthritic changes in the cartilage. The cartilage scoring was performed according to the OARSI scheme described in [12]. Figure 1a(E) depicts examples of the obtained histological images for OARSI scores 1–5. Additionally, two serial slices of 4 μm for each sample were stained with safranin orange-fast green for further investigation of the degree of OA.

### 2.4. Signal Processing and Feature Extraction

The vibroacoustic signal shown in Figure 1a(D) comprises transient responses from collisions between the instrument tip and the non-homogeneous tissue surface, and stationary signal segments from smooth instrument–tissue friction in homogeneous surface areas. The primary hypothesis is that characteristic features related to the cartilage’s structural and surface properties can be extracted. Figure 1b depicts the processing steps applied for feature extraction. The signals were de-trended via band-pass filtering using Discrete Wavelet Transformation (DWT) with Daubechies 4 mother wavelet and a passband of approximately $f_{passband}\approx 5$ Hz to 7 kHz. The band was adopted from our previous vibroacoustic characterization work [35]. The DWT removes the low-frequency baseline drift below this range. The upper limit of 7 kHz retains the informative spectral content of the instrument–tissue interaction within the 16 kHz sampling bandwidth. The individual palpation experiments within one measurement were manually annotated and segmented for an independent analysis. The Continuous Wavelet Transformation (CWT) Spectrum was computed using a Morse mother wavelet because it is suitable for signals containing transient responses.

Three indicators in the frequency and time-frequency domain were derived as described in [35]: (1) The **Instantaneous Dominant Frequency (IDF)** is defined as the time-variant frequency component dominating the signal. It is derived from the CWT wavelet scale and computed per time instant *t* as the frequency of maximal spectral energy. Changes in IDF can be related to the stability of the signal and, therefore, to the surface’s homogeneity. (2) The **IDF’s Histogram**, which is the probability distribution of IDF values, represents the dominant spectral bands regarding energy. It is less affected by disparate energy intensities of interactions within one signal but does not allow assessing the energy distribution over all frequency components present. (3) The **CWT-based Stationary (averaged) Spectrum** represents all frequency components mainly present in the signal. The energy distribution of this spectrum appears to be strongly related to the hardness or softness of a tissue [35]. The signal analysis was performed using MATLAB R2022a. 26 features were extracted from the indicators. 9 energy-related features $F_{1-9}$ discussed in [35] were extracted following a qualitative analysis. In addition, the features $F_{10-17}$ were extracted as non-normalized representations of $F_{2-9}$. Further, 4 statistical features, $F_{18-21}$, and 5 dynamical features, $F_{22-26}$, were added. Table 2 summarizes the features, their basic indicator, and a short description. For the features $F_{2-17}$, four subbands were defined according to [35]: very low frequencies (VLF) =1–116 Hz, low frequencies (LF) =116–286 Hz, middle frequencies (MF) =286–1300 Hz, and high frequencies (HF) =1300–7000 Hz. The 26-dimensional feature set and the wavelets were adopted from our previous work [35]: the Daubechies-4 wavelet for de-trending and the Morse wavelet for the CWT, both suited to signals that combine transient collisions with stationary friction. The feature groups are intentionally complementary—covering spectral energy distribution, dominant-frequency statistics, and surface dynamics—rather than mutually independent. Thus, no prior assumption on the most discriminative feature is required. The 26 features were adopted unchanged from this earlier work to keep the results comparable. Some of them are correlated by construction, for example the normalized and non-normalized sub-band energies $F_{2-9}$ and $F_{10-17}$. As all features are standardized before classification, this redundancy mainly affects interpretability rather than the classification itself. A dedicated feature-selection step to remove the collinearity, particularly relevant for the distance-based kNN, is left for future work.

$F_{1}$, the total energy of the stationary spectrum, reflects tissue hardness, while $F_{2-5}$ are its band energies normalized to $F_{1}$. Because interactions with hard or soft tissue will reflect in different bands, they help to differentiate between comparably dissimilar tissues. The non-normalized counterparts $F_{10-13}$ are more affected by the interaction intensity and the individual volunteer. Analogously, $F_{6-9}$ and $F_{14-17}$ are the normalized and absolute band energies of the IDF histogram. Based on the dominating frequency per time instant, these features contain information more focused on the dominating interactions concerning intensity. The statistical features $F_{18-21}$ capture the range and stability of the dominant frequency, e.g., the variance $F_{21}$ as a measure of surface homogeneity. The dynamic features $F_{22-26}$ count IDF changes within the time intervals very short (vsh,≤0.625 ms) to very long (vlo,≥62.5 ms), normalized to the signal length. They thus describe surface smoothness or roughness: a smooth surface produces few changes (mainly $F_{26}$), whereas alternating smooth and rough areas excite $F_{22-24}$.

### 2.5. Dataset Generation

The analysis comprised 136 specimens from 41 patients (41 knees). A total of 435 computerized palpation measurements were obtained, resulting in a dataset of 8700 individual palpation experiments. The OARSI scores were used as ground truth for labeling three classes. According to clinical experts, discrimination between higher (OARSI ≥2.5) and lower grades of cartilage degeneration (OARSI ≤2.0) would provide a sufficient tool for assisting a decision between UKA and TKA. Accordingly, three classes were defined: The **class 1 of OARSI ≤2.0** represents healthy to still acceptable cartilage; the **class 2 of OARSI ≥2.5** represents degenerated osteoarthritic cartilage; and the **class 3 of OARSI =6.0** represents loss of cartilage (bone). As the OARSI score is assigned in discrete steps of 0.5, the classes “≤2.0” and “≥2.5” are contiguous and jointly exhaustive of the cartilage grades. No score lies in between. The binary cartilage task (classes 1 and 2) therefore covers grades 0.5–2.0 vs. 2.5–5.0, while bone (OARSI 6.0) forms the separate third class. Table 3 summarizes the dataset, including the distribution of OARSI scores for the later classification.

Because the palpations of a given specimen are not statistically independent, the classification was validated with grouped cross-validation (CV). All palpations of a specimen and of a patient are kept together within a single fold. Two complementary grouping schemes were used. The first is a patient-level Leave-One-Patient-Out cross-validation over the 41 patients. The second is a specimen-level stratified grouped cross-validation over the 136 specimens. Both yielded equivalent results, and the specimen-level scheme is used for the figures. The feature vector ${\vec{F}}_{1-26}$ was computed for every palpation and used for the classification step described in the next section. Two test cases of different clinical use scenarios were defined: In **Test Case A**, each palpation experiment is classified individually, representing a single-palpation cartilage assessment. In **Test Case B**, an averaged feature vector of all 20 palpation experiments of a measurement was computed and classified. It represents an assessment using a full measurement of 20 palpations to increase the robustness against minor variations in the palpation location.

### 2.6. Classification

Support Vector Machine (SVM) [37] and k-Nearest-Neighbors algorithm (kNN) [38] were selected for the classification. SVM is a parametric method that aims to find a hyperplane in the N-dimensional space that best separates the classes in a dataset. The regularization parameter *C* (controlling the cost of a misclassification during training) and the kernel function (used to transform the input data) were optimized in the training process. kNN is a non-parametric method that classifies a data point using majority voting based on the class labels of the *k* nearest neighbors in the feature space. Two hyperparameters, the number of neighbors *k* and the distance metric (used to determine the similarity between data points), were optimized during training. Hyperparameters were optimized by grid search within a nested inner grouped cross-validation performed on the training folds only, so that the held-out specimen/patient never influenced model selection [39,40]. The searched grids were C∈{0.01,0.1,1,10,100} (logarithmic, factor-of-ten steps) for the SVM and k∈{5,15,29,45,75} for the kNN. The SVM used a Gaussian kernel for the three-class task and a quadratic kernel for the binary task, and the kNN used the city-block distance. The outer loop consisted of 41 folds for the patient-level Leave-One-Patient-Out scheme and of five stratified folds for the specimen-level scheme. The inner grid search used a grouped three-fold cross-validation and the classification accuracy served as the selection criterion. All 26 features were standardized to zero mean and unit variance. The scaling parameters were estimated on the training fold only and then applied to the held-out data, so that no information from the test fold entered the preprocessing.

Their performance was tested based on the defined test cases A and B. The optimization, training, and test processes were conducted separately for the entire dataset and a subset comprising only classes 1 and 2. To quantify statistical uncertainty, 95% confidence intervals (CI) were obtained for all metrics by a patient-cluster bootstrap with 2000 replicates. Whole patients rather than individual palpations were resampled for both grouping schemes. In this way, the intervals reflect the number of statistically independent units (41 patients) and are not narrowed by the within-patient correlation of repeated palpations. The confusion matrices were computed along with the measurement metrics accuracy (ACC), sensitivity (SE), and specificity (SP) to assess the classifier’s performance. Further, the misclassification rate per OARSI score was calculated and visualized to analyze the misclassification distribution.

Based on the good results for the discrimination of class 3 (bone), the classifiers were further assessed for the binary discrimination of the cartilage classes 1 and 2. Across the grouped cross-validation, every palpation and measurement of the binary task is evaluated once as a held-out sample, yielding 7660 out-of-fold classifications for case A and 383 for case B. For this binary classification, the receiver operating characteristic curve (ROC) was computed in addition per classifier and test case. The ROC can be used in medical research to evaluate the performance of a diagnostic test by illustrating the trade-off between SE and SP for different test thresholds [41,42]. A perfect test with SE=SP=1 would yield a point in the upper left corner of the ROC space, while a random guess would give a point along the diagonal so-called line of no-discrimination [43]. The area under the ROC (AUC) is a quantitative measure of a classifier’s ability to distinguish between the two classes, with higher values indicating better performance and AUC≥0.5 performing better than random chance [44]. As a second metric, Youden’s index represents the maximum height of a point above the line of no-discrimination and was used as the selection criterion for the optimum cut-off point with respect to the varied discrimination threshold [45,46].

## 3. Results and Discussion

### 3.1. Classification Results

In brief, under grouped cross-validation, bone was separated from cartilage reliably (84–91% recall), healthy cartilage (OARSI ≤2.0) was identified with moderate sensitivity (74–80%), whereas the low specificity for degenerated cartilage (OARSI ≥ 2.5) (40–57%) remained the principal limitation. The detailed metrics with 95% confidence intervals are reported below.

**Figure 2:**
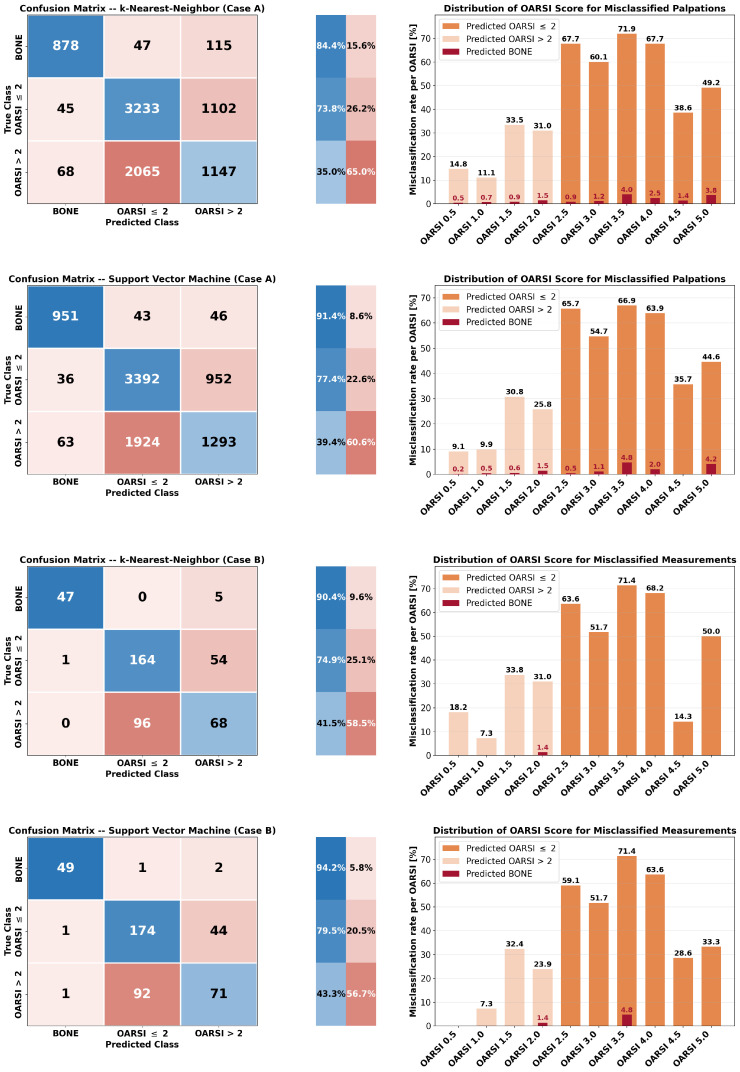
Confusion matrices for test case A and test case B, each for kNN (**top**) and SVM (**bottom**), with the row-normalized recall in the adjacent column. The distributions of misclassification rates per individual OARSI score are shown on the (**right**), revealing that degenerated cartilage (OARSI ≥ 2.5) is predominantly misclassified as well-preserved cartilage across all grades, whereas intact cartilage (OARSI 0.5–1.0) and bone are classified most reliably. Test case A—classification per palpation experiment (kNN **top**, SVM **bottom**). Test case B—classification per measurement, average of the 20 palpations (kNN **top**, SVM **bottom**).

Table 4 summarizes the overall ACC, SE, and SP for validation with the test set and the used hyperparameters, together with the AUC and Youden’s index of the ROC analysis for the binary task. The equivalent patient-level Leave-One-Patient-Out results are reported in Appendix A (Table A1). The confusion matrices are visualized in Figure 2. A paired Wilcoxon signed-rank test on the per-patient accuracies compared the two classifiers (Table A2). The SVM significantly outperforms the kNN in the three-class task (p<0.01). For the binary task, it outperforms the kNN in the per-palpation case A (p=0.04). The per-measurement aggregation of test case B significantly improves the SVM (p<0.05) but not the kNN. The remaining differences between the test cases lie within the confidence intervals. For classes 1–3 and test case A, both classifiers show a moderate performance with an ACC of 0.60 (kNN) and 0.65 (SVM), improving to 0.64 and 0.68 for test case B. The sensitivity for healthy cartilage (class 1) is moderate (74–80%), whereas the specificity for degeneration is low (51–57%). For the subset of classes 1 and 2, the ACC lies between 0.60 and 0.65, with SE of 76–80% but SP of only 37–45%. All values carry wide confidence intervals (Table 4), reflecting the limited number of independent patients.

The performance difference for the subset can be explained by the strong results for the excluded class 3 reflected in the confusion matrices. The discrimination of bone shows a good recall of 84–91% for SVM and kNN. Compared to cartilage, the different surface characteristics, hardness, and non-damping character facilitate discrimination due to significantly different resulting vibroacoustic signals [35]. Differentiating bone versus soft tissue could be highly relevant for applications such as robotic spine surgery, where new sensing approaches could enable a higher degree of automation [47]. While accelerometer-based sensing [48] or contact microphones for bone acoustic emissions [49] were used in this context, a vibroacoustic sensing setup such as that used in this study has not been presented.

Besides bone, Figure 2 shows that cartilage of OARSI ≤2 can be identified with moderate SE between 74% and 80%. In contrast, the recall for cartilage of OARSI ≥2.5 is low (35–39% under grouped cross-validation), i.e., the majority of degenerated specimens are misclassified as well-preserved cartilage (Figure 2). This limited discrimination of degeneration is reflected in the low overall SP shown in Table 4. The low SP would cause an unacceptably high false positive rate for cartilage of OARSI ≥2.5 in a clinical test for well-preserved cartilage. However, the presented approach for cartilage assessment could be one important source of information, out of multiple sources, that facilitates a treatment decision. Consequently, the method is not intended as a stand-alone diagnostic test for advanced degeneration but as a complementary, low-cost source of intraoperative information. Its high sensitivity for well-preserved cartilage and reliable bone detection are most relevant for UKA/TKA decision support, where missing well-preserved cartilage is more costly than an occasional false positive. The difference could be explained by a lower variance in the surface properties of the less degenerated cartilage of class 1. This possibly mitigates influences of the experimental setup, such as minor deviations of the palpation path. A resulting more homogeneous dataset of signals and extracted features could explain the better performance. This hypothesis is supported by the low misclassification rate for OARSI 0.5–1.0 shown in Figure 2(right). Specimens of this score represent intact cartilage expected to show the highest homogeneity of tissue and surface characteristics. Further, this indicates that intact cartilage could potentially be detected even better.

In contrast, cartilage of OARSI ≥2.5 shows higher degeneration, reflected in a greater variance in surface characteristics, thickness, and structural integrity (Figure 1a(E)). While the aim is to extract and use this inherent information for classification, grouping OARSI 2.5–5.0 in class 2 likely results in a less homogeneous dataset, which could explain this class’s lower performance. This explanation is supported by the misclassification rates shown in Figure 2.

Class imbalance further contributes to the limited detection of degeneration. The three classes are unequally populated, with 4380 palpations for healthy cartilage (OARSI ≤2.0), 3280 for degenerated cartilage (OARSI ≥ 2.5), and 1040 for bone. The degenerated class is additionally the most heterogeneous, spanning grades 2.5–5.0. Under grouped cross-validation, the classifiers therefore tend to favor the majority, well-preserved class, which manifests as the high sensitivity for healthy cartilage but low specificity for degeneration reported above. Re-training the SVM with balanced class weights was evaluated but did not materially improve the specificity for degenerated cartilage, indicating that the limitation stems primarily from intrinsic feature overlap between moderately and severely degenerated tissue rather than from imbalance alone. This should be addressed in future work through targeted acquisition of additional degenerated specimens and degeneration-aware feature design. OARSI scores ≤ 1.0 show the lowest misclassification (≈10%), rising to ≈25% for scores 1.5–2.0. Within class 2, the rate peaks at ≥54.5% for OARSI 2.5 before fluctuating up to OARSI 4.5. As this peak does not appear for the adjacent OARSI 2.0, it likely reflects the under-representation of OARSI 2.5 in class 2 (about one third of the data of OARSI 2.0 and 3.0, Table 3) rather than an inherent threshold problem. A balanced, uniformly distributed dataset—difficult to obtain from ex vivo surgical tissue—would be a prerequisite for alternative class definitions or additional classes, e.g., grouping OARSI scores by biological and morphological rather than purely clinical aspects. None of the scores are prone to confusion with bone (only a marginal increase for OARSI 5.0 in test case A), and the averaging in test case B reduces inter-specimen and inter-OARSI variance unevenly, benefiting mainly scores 0.5–1.0. Such an imbalance is largely inherent to ex vivo sampling during arthroplasty, where higher degeneration grades dominate the harvested tissue. A prospective, multi-center collection would be required to balance the lower grades and is regarded as essential future work.

In addition to the quantity and balance of the data, the statistical dependence between palpations of the same specimen must be discussed. Although the manual palpation introduces variance in contact angle, force, path and velocity between the 20 experiments of a measurement, all palpations of a specimen share the same tissue and label and are therefore not statistically independent. The grouped cross-validation accounts for this by keeping all palpations of a specimen and of a patient within a single fold, so that generalization is estimated on entirely unseen specimens and patients. That the extracted features were shown to be largely independent of the palpating subject [35] is relevant for generalization across operators but does not remove this within-specimen dependence.

The palpations can also be aggregated to the specimen level. A single majority vote over all palpations of a specimen raises the three-class accuracy to 0.728 (95% CI [0.64, 0.80]) for the SVM and to 0.713 [0.63, 0.79] for the kNN. At this level, the sensitivity for well-preserved cartilage reaches 0.88 (SVM) and 0.87 (kNN). The specificity for degeneration reaches 0.57 and 0.55, respectively. A single patient-level aggregate is not meaningful, as each patient contributed specimens of different OARSI grades and therefore has no single reference label.

### 3.2. Binary Cartilage Classification as a Diagnostic Test

**Figure 3:**
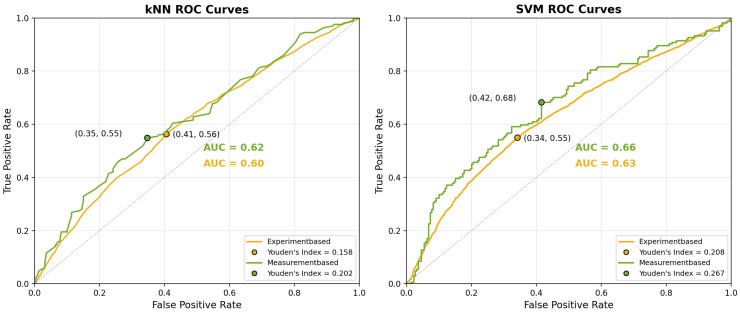
ROC curves for the binary cartilage classification (class 1, OARSI ≤2.0 vs. class 2, OARSI ≥2.5) for kNN (**left**) and SVM (**right**), obtained from the specimen-level grouped cross-validation. Curves are shown for the experiment-based test case A and the measurement-based test case B. The marked operating points correspond to the maximum Youden’s index. The AUC values match Table 4. The equivalent patient-level values are given in Appendix A (Table A1).

As the binary classification of the cartilage classes 1 and 2 can be interpreted as a diagnostic test for well-preserved cartilage, the ROC curves shown in Figure 3 allow a visual evaluation of the classifiers and test cases. For a quantitative comparison, the AUC was computed and is reported in Table 4. While the results are in a similar range, the SVM provides the best result with AUC=0.66 (95% CI 0.56–0.76), followed by the kNN with AUC=0.62 (0.54–0.71), both for the measurement-based test case B. The corresponding Youden’s indices are 0.27 (SVM) and 0.20 (kNN). The associated operating points labelled in the ROC curves represent the optimal threshold for the classifiers given an identical weighting of both misclassification errors. The moderate AUC values are consistent with the limited specificity discussed above: the approach reliably ranks well-preserved above degenerated cartilage but does not yet provide the discriminative power required for a stand-alone diagnostic decision. It therefore appears most promising as one complementary source of information within a multi-parameter intraoperative assessment.

Several limitations should be emphasized. First, the reported performance was obtained by internal grouped cross-validation. Although both patient-level and specimen-level schemes were used and agreed, no independent external cohort was available, and the data originate from a single center. Prospective multi-center validation is required before any clinical claim. Second, the cohort is modest (41 patients, 136 specimens) and imbalanced across degeneration grades, which widens the confidence intervals, particularly for the binary AUC. Third, data were acquired by six trained operators. Measurements from several operators per specimen are pooled, so that the reported grouped performance already integrates over operator variability. A dedicated inter-operator (leave-one-operator-out) analysis was beyond the scope of this feasibility study and is a valuable direction for future work. These points reinforce that the method is presented as a proof of concept and complementary information source rather than a validated diagnostic tool.

### 3.3. Experimental Setup

The experimental setup aimed to resemble the situation during arthroplasty. The palpation instrument is commonly used in arthroscopic surgery and could be integrated easily into the workflow. However, future studies must address influence factors such as flushing of the surgical site or contact of the instrument with other tissues during palpation. Besides the acquisition setup, the general limitation of the OARSI score as ground truth must be discussed. While the score was found to have excellent intra- and inter-observer reproducibility with less dependency on the observer experience [50], it must be noted that only two experienced clinical researchers graded the specimen in this study. The average bicondylar knee width of 71.23 mm ±7.53 mm provides space for multiple possible locations for the degeneration of cartilage [51]. While the cartilage specimens were palpated over the entire length of 10 mm, histologic samples were taken from a central part of the specimen via micro-sectioning. This results in sections of 4 μm as the basis for the OARSI grading. Consequently, the OARSI score must be considered a spatial random sample of the specimen’s cartilage quality, which may not be identical to the quality along the palpation path. Given the different scales of both methods, the obtained score is assumed to represent the entire specimen for the scope of this study. Further, the OARSI score is based on individual markers identified in the histologic image and does not necessarily capture the overall characteristics of the cartilage or its surface. Particulars of the image can significantly downgrade the score while the surrounding cartilage can be intact. In contrast, the computed features intend to capture the characteristics of the full palpation path, potentially making them less sensitive to individual discontinuities such as fissures. However, no better and widely accepted ground truth for the cartilage quality is available. As histology and palpation were not spatially co-registered, the graded section and the acquired signal may not originate from exactly the same location. A denser, registered sampling of several sections per specimen would tighten this ground truth in future studies. Although ex vivo harvesting is not itself a clinical procedure, the acquisition was deliberately designed to mirror the intraoperative situation: it uses the same arthroscopic palpation probe, a comparable contact angle and manual actuation. Consequently, the approach is expected to transfer to open UKA/TKA and, after adaptation for joint-space flushing and port access, to arthroscopy. The present ex vivo setting should therefore be read as a controlled feasibility step toward in vivo application.

Concerning specimen preparation, the influence of PBS on the biomechanical properties of cartilage is not well understood yet. Some studies suggest that PBS can alter the tissue’s water content and mechanical properties over time [52,53]. However, the studies used tendon or previously frozen cartilage tissue samples to assess the effect, or investigated the effect after 8 h conservation in PBS. In this study, specimens remained in PBS for an average of 49 min and a maximum of 2 h 10 min. Because the effects seem dependent on the exposure time, the influence on this study is considered minimal but should be addressed in future work. Further, the mechanical stress caused by the computerized palpation must be discussed. 40–80 palpations were performed before histologic sampling but without applying excessive contact forces. Given the physiologic loads that cartilage must resist during normal articulation, no significant influence of the palpation on the cartilage quality is assumed. While it was shown in [35] that the influence of the palpating volunteers can be considered low, a more controlled acquisition in terms of palpation velocity, contact angle, and contact force may improve the results. This could be achieved in a robot-assisted surgery setup. With the Mako system from Stryker (Kalamazoo, MI, USA) and the ROSA knee system from Zimmer Biomet (Warsaw, IN, USA) already widely adopted for UKA/TKA, integration of the presented approach appears feasible.

### 3.4. Signal Processing

The computed features provide a solid representation of the palpation signal. For bone, the discriminative power is sufficient for a reliable detection among softer cartilage tissue. Although the data for this class was comparably low, significant differences in the mechanical properties facilitated a very good result. As presented in [35], harder and less damping tissues cause less energy to be absorbed during the interaction. This is reflected in the palpation signal’s higher energy and frequencies. In contrast, one primary function of the cartilage is to absorb loads, resulting in lower energies and frequencies in the signal. Such differences in the signal characteristic can be captured by the primarily energy-related features used in this study. Analyzing the features regarding their discriminative power would be a reasonable next step. For example, identifying the features that contribute the most to bone classification could facilitate less computationally intensive real-time identification. Such real-time detection could be of great interest for applications that require manipulating bone and protecting surrounding softer tissues or vice versa.

The proposed features are less significant for the differentiation of the cartilage-related classes 1–2. The increase in misclassification for OARSI scores 2.5–4.0 indicates that the features do not sufficiently capture the changes in tissue characteristics associated with higher levels of degeneration. The energy-related features seem insufficient for a reliable classification because the mechanical properties are more similar for the different degrees of degeneration compared to bone. While $F_{22-26}$ try to cover the roughness or smoothness of the surface, other important characteristics are not considered. When one thinks of the palpation path as a profile, the signal follows this path with excitations due to peaks, valleys, or discontinuities. This suggests complementing them with morphological features of the palpation path such as waviness, peak density, peak spacing, or valley depth. Empirical Mode Decomposition [54], with its better low-frequency selectivity and its handling of non-linearities, together with envelope- and auto-regressive modeling-based methods [55], appear better suited for this. Moreover, the present features focus on the continuous-movement part of the signal. The on- and offset of contact, where the instrument’s natural frequency is modulated, should additionally be exploited, as its non-linear dynamics presumably carry complementary information.

## 4. Conclusions

This study explored the potential of computerized vibroacoustic palpation to assess the degree of cartilage degeneration in 136 human ex vivo specimens. Bony tissue could be discriminated from cartilage reproducibly (recall 84–91%). Such an identification may be helpful not only in knee joints but also, e.g., in identifying the bony surface in spinal interbody fusions to ensure the absence of intervertebral body tissue, to identify the complete removal of soft tissues of the bone-implant interface in the femoral canal during revision surgery, or to ensure complete bone cement removal in a cemented revision. For cartilage, the dataset’s composition and size did not allow division into classes of degeneration levels similar to the OARSI scores. Therefore, the cut-offs for determining classes were set as bone, OARSI grade ≥ 2.5, and OARSI grade ≤ 2.0. The classification approach performs well in identifying less degenerated cartilage with OARSI scores ≤ 2.0. However, the low SP for the OARSI scores ≥ 2.5 reflects one of the study’s main limitations, and the reliable grading of cartilage degeneration remains an open challenge. Interpreted as a diagnostic test, the binary cartilage classification reached an AUC of up to 0.66, indicating discriminative ability only modestly above chance and underlining that the approach is currently best suited as a complementary, rather than stand-alone, decision aid. A more extensive dataset, extracting additional features, and a more standardized palpation process, e.g., in a robot-assisted surgery setup, could certainly improve cartilage results. Further investigations are needed to assess signal acquisition’s reproducibility, individual features’ discriminative power, and integration into the surgical workflow.

To our knowledge, this is the first ex vivo study to investigate tissue–instrument interactions as a source of information for surgical-data science and a potential live and in vivo assessment of cartilage degeneration. Methodologically, the present work goes beyond earlier vibroacoustic tissue-type discrimination [35] by grading the severity of degeneration within a single tissue type and by framing the binary cartilage decision as a diagnostic test with a full ROC analysis. From a clinical perspective, the approach may provide complementary information that could support, rather than replace, intraoperative decision-making. Compared to technologies such as NIRS, OCT, or IT-AFM, a surgeon could easily obtain results without extensive training and with a cost-effective tool. Further, the tool does not harm healthy tissue during measurements such as biopsy or indentation-based methods. In summary, instrument–tissue interactions may provide complementary information in the clinically relevant use case of arthroplasty, although reliably grading the severity of degeneration remains challenging.

## Figures and Tables

**Table 1:** Summary of the patient population included in this study.

N	Sex	Age [yr]	Weight [kg]	BMI	UKA/TKA
15	female	69.3±10.5	85.4±10.6	31.3±4.1	5/10
26	male	64.4±8.3	98.2±21.5	31.1±6.4	9/17

**Table 2:** Summary of energy-related (yellow), statistical (green), and dynamical (blue) features. Spectral energies (SE) and histogram’s energies (HE) correspond to the sub-bands VLF=1–116 Hz, LF=116–286 Hz, MF=286–1300 Hz, HF=1.3–7.0 KHz. Dynamic frequency changes (DFC) correspond to the time intervals vsh = ≤0.625 ms, sho=0.625–6.25 ms, med=6.25–31.25 ms, lon=31.25–62.5 ms, vlo = ≥62.5 ms.

Feature	Basis	Name	Description
$F_{1}$	Stationary (averaged) CWT-Spectrum	TE	total energy of the spectrum
$F_{2-5}$		${\hat{SE}}_{VLF\ldots HF}$	normalized spectral energies in sub-bands VLF…HF
$F_{10-13}$		$SE_{VLF\ldots HF}$	spectral energies in sub-bands VLF…HF
$F_{6-9}$	IDF’s Histogram	${\hat{HE}}_{VLF\ldots HF}$	normalized histogram’s energies in sub-bands VLF…HF
$F_{14-17}$		$HE_{VLF\ldots HF}$	histogram’s energies in sub-bands VLF…HF
$F_{18}$	CWT Spectrum	$F_{max}$	frequency of maximal excitation
$F_{19}$	IDF	$DF_{max}$	maximal dominant frequency
$F_{20}$		$DF_{min}$	minimal dominant frequency
$F_{21}$		$DF_{var}$	variance in dominant frequency
$F_{22-26}$		$DFC_{vsh\ldots vlo}$	IDF changes in corresponding time intervals vsh…vlo

**Table 3:** Composition of the dataset, comprising 136 cartilage specimens from 41 patients (41 knees). Classification was validated with patient- and specimen-level grouped cross-validation (Section 2.6).

OARSI	Knees	Specimens	Measurements	Palpations	Class	Class Total
0.5	6	6	22	440	1	4380
1.0	15	17	55	1100		
1.5	18	22	71	1420		
2.0	16	24	71	1420		
2.5	7	7	22	440	2	3280
3.0	14	18	58	1160		
3.5	7	7	21	420		
4.0	11	13	44	880		
4.5	2	2	7	140		
5.0	4	4	12	240		
6.0	16	16	52	1040	3	1040

**Table 4:** Results for kNN and SVM under specimen-level grouped cross-validation with nested hyperparameter tuning (patient-level Leave-One-Patient-Out gives equivalent values). Point estimates are shown with 95% CI (patient-cluster bootstrap) in brackets. The values correspond to accuracy (ACC), sensitivity (SE, recall of class 1), and specificity (SP, of class 1), as well as the area under the ROC (AUC) and Youden’s index for the two-class task. Hyperparameters (*C*, *k*) were tuned per fold on the training data. The SVM used a Gaussian (three-class) or quadratic (two-class) kernel, and the kNN used the city-block distance.

Classes	Classifier	Kernel/dist.	Test Case	Grouped CV			ROC
				ACC	SE	SP	AUC (Youden)
**1–3**	**SVM**	gaussian	A	0.648 [0.58, 0.71]	0.774 [0.71, 0.83]	0.545 [0.47, 0.62]	−
			B	0.676 [0.60, 0.75]	0.795 [0.72, 0.86]	0.569 [0.47, 0.67]	−
	**kNN**	city block	A	0.604 [0.54, 0.65]	0.738 [0.69, 0.78]	0.511 [0.43, 0.59]	−
			B	0.641 [0.57, 0.71]	0.749 [0.67, 0.83]	0.556 [0.46, 0.66]	−
**1–2**	**SVM**	quadratic	A	0.622 [0.56, 0.68]	0.787 [0.73, 0.83]	0.402 [0.33, 0.47]	0.63 [0.55, 0.69] (0.21)
			B	0.648 [0.57, 0.72]	0.799 [0.72, 0.87]	0.445 [0.33, 0.56]	0.66 [0.56, 0.76] (0.27)
	**kNN**	city block	A	0.599 [0.54, 0.66]	0.768 [0.71, 0.82]	0.372 [0.30, 0.45]	0.60 [0.53, 0.67] (0.16)
			B	0.614 [0.54, 0.69]	0.758 [0.68, 0.83]	0.421 [0.32, 0.52]	0.62 [0.54, 0.71] (0.20)

## Data Availability

The data that support the findings of this study are not openly available for reasons of sensitivity and protection of the privacy of the research participants and are available from the corresponding author upon reasonable request.

## Appendix A. Additional Numerical Results

### Appendix A.1. Patient-Level (Leave-One-Patient-Out) Results

Table A1 reports the numerical results of the patient-level Leave-One-Patient-Out cross-validation, mirroring Table 4. The two grouping schemes agree to within the 95% confidence intervals.

**Table A1:** Patient-level Leave-One-Patient-Out results, mirroring Table 4. Point estimates are shown with 95% patient-cluster-bootstrap CI in brackets. AUC and Youden’s index are given for the binary task.

Classes	Classifier	Kernel/dist.	Test Case	ACC	SE	SP	AUC (Youden)
**1–3**	**SVM**	gaussian	A	0.649 [0.59, 0.71]	0.795 [0.74, 0.85]	0.526 [0.45, 0.61]	−
			B	0.694 [0.62, 0.76]	0.813 [0.74, 0.88]	0.593 [0.49, 0.70]	−
	**kNN**	city block	A	0.621 [0.55, 0.68]	0.773 [0.71, 0.83]	0.505 [0.42, 0.59]	−
			B	0.637 [0.56, 0.70]	0.763 [0.68, 0.84]	0.532 [0.43, 0.63]	−
**1–2**	**SVM**	quadratic	A	0.614 [0.54, 0.68]	0.793 [0.73, 0.85]	0.375 [0.29, 0.46]	0.61 [0.54, 0.69] (0.19)
			B	0.645 [0.56, 0.73]	0.799 [0.71, 0.87]	0.439 [0.32, 0.57]	0.66 [0.55, 0.76] (0.25)
	**kNN**	city block	A	0.594 [0.51, 0.66]	0.789 [0.72, 0.85]	0.333 [0.25, 0.42]	0.59 [0.50, 0.67] (0.14)
			B	0.614 [0.52, 0.69]	0.772 [0.69, 0.84]	0.402 [0.29, 0.52]	0.60 [0.50, 0.70] (0.18)

### Appendix A.2. Statistical Comparison of Classifiers and Test Cases

Table A2 reports the paired Wilcoxon signed-rank tests that underlie the comparison of classifiers and test cases in Section 2.6. For each of the 41 patients, the classification accuracy over that patient’s palpations was computed from the out-of-fold predictions, giving 41 paired values per comparison. A two-sided Wilcoxon signed-rank test was then applied to these paired per-patient accuracies. This non-parametric test does not assume a normal distribution and treats the patients as the independent units. The same tests applied to the specimen-level scheme yield equivalent conclusions.

**Table A2:** Paired Wilcoxon signed-rank tests on the per-patient accuracies (n=41 patients). Values are the mean per-patient accuracies of the two compared settings and the two-sided *p*-value.

Task	Comparison	Test case	Mean 1	Mean 2	*p*
**1–3**	SVM vs. kNN	A	0.637	0.608	<0.001
	SVM vs. kNN	B	0.686	0.627	0.006
	A vs. B (SVM)	–	0.637	0.686	0.007
	A vs. B (kNN)	–	0.608	0.627	0.375
**1–2**	SVM vs. kNN	A	0.604	0.586	0.036
	SVM vs. kNN	B	0.646	0.603	0.191
	A vs. B (SVM)	–	0.604	0.646	0.038
	A vs. B (kNN)	–	0.586	0.603	0.432
